# Hallux valgus angle as main predictor for correction of hallux valgus

**DOI:** 10.1186/1471-2474-9-70

**Published:** 2008-05-15

**Authors:** Axel R Deenik, Enrico de Visser, Jan-Willem K Louwerens, Maarten de Waal Malefijt, Frits F Draijer, Rob A de Bie

**Affiliations:** 1 Dept. of Orthopedic Surgery, Hospital Bronovo, the Hague, the Netherlands; 2 Dept. of Orthopedic Surgery, Sint Maartenskliniek, Nijmegen, the Netherlands; 3 Dept. of Orthopedic Surgery, UMC St. Radboud, Nijmegen, the Netherlands; 4 Dept. of Orthopedic Surgery, Maasland Hospital, Sittard, the Netherlands; 5 Dept. of Epidemiology, University of Maastricht, Maastricht, the Netherlands

## Abstract

**Background:**

It is recognized that different types of hallux valgus exist. Classification occurs with radiographic and clinical parameters. Severity of different parameters is used in algorithms to choose between different surgical procedures. Because there is no consensus about each parameter nor their cut-off point we conducted this study to analyze the influence of these variables on the postoperative hallux valgus angle.

**Methods:**

After informed consent 115 patients (136 feet) were included. Bunionectomy, osteotomy, lateralization of the distal fragment, lateral release and medial capsulorraphy were performed in all patients. Data were collected on preoperative and postoperative HVA, IMA and DMAA measurements. Forty cases were included since our findings in a previous article [1], therefore, current data concern an expanded study group with longer follow-up and were not published before. At least two-year follow-up data were evaluated with logistic regression and independent t-tests.

**Results:**

Preoperative HVA was significant for prediction of postoperative HVA in logistic regression. IMA and DMAA were not significant for prediction of postoperative HVA in logistic regression, although they were significantly increased in larger deformities. In patients with preoperative HVA of 37 degrees or more, satisfactory correction could be obtained in 65 percent. The other nine of these 26 patients developed subluxation.

**Conclusion:**

The preoperative HVA was the main radiological predictor for correction of hallux valgus, correction rate declined from preoperative HVA of 37. IMA and DMAA did have a minor role in patients with preoperative HVA lower than 37 degrees, however, likely contributed to preoperative HVA of 37 degrees or more.

## Background

About 120 different operation techniques are described for the treatment of symptomatic hallux valgus. However, these procedures result in a 10 percent recurrence rate [2]. Radiographic and clinical parameters, like hallux valgus angle (HVA), intermetatarsal angle (IMA) and distal metatarsal articular angle (DMAA) and tarsometatarsal (TMT) hypermobility, have been developed to identify different types of hallux valgus. Severity of each parameter is based on cut-off points and used in algorithms to choose between different surgical procedures [3,4]. Radiographic cut-off points lie between 20–40 degrees for HVA, 11–20 degrees for IMA and 10–15 degrees for DMAA.

Evidence of algorithms are derived from few retrospective studies [5]. Outcomes of other studies do not support the validity of individual features such as TMT hypermobility, IMA [6] and DMAA [7]. Randomized controlled trials show no difference in correction between Lapidus and distal osteotomy nor shaft and distal osteotomy [6,1]. It is questionable if these radiological parameters need to be corrected, or if the cut-off points used for the HVA, IMA or DMAA are correct. Therefore, the influence of parameters on the hallux valgus angle should be evaluated and algorithms should be tested.

In analyzing parameters it is necessary to choose an outcome parameter. AOFAS [8] score is used in evaluating results, the validity is limited because of overemphasis on evaluating pain [9]. Meta-analysis showed that rating scales lack in reporting the results of clinical studies [10].

Because the hallux valgus angle is the main derivative of hallux valgus, the HVA is an objective parameter for evaluation of correction obtained through surgery. Algorithms are justified for more aggressive surgery in moderate and severe hallux valgus deformities to obtain better correction of HVA. However, it is recognized that clinical and radiological parameters are lacking in evaluating surgical results [9,10].

The purpose of this study is to evaluate the influence of parameters before surgery on the HVA after surgery.

## Methods

Between August 1999 and September 2004 141 feet in 120 consecutive patients, including bilateral cases were randomized. Inclusion criteria were patients with a painful bunion and hallux valgus between 18 and 65 years of age and an adequate range of movement. Exclusion criteria were patients with rheumatoid arthritis, failed previous hallux valgus surgery and symptomatic and/or radiological arthritis of the MTP joint. Three cases were excluded after a Scarf osteotomy and one case after a Chevron osteotomy because they were not operated according to study protocol. One case with Scarf osteotomy refused to attend follow-up because of psychiatric problems and debilitating chronic regional pain syndrome (CRPS). 70 Chevron osteotomies and 66 Scarf osteotomies were seen at follow-up 2,4 years (range 23–39 months) after surgery. Osteotomy, lateralization of the distal fragment, bunionectomy, transarticular lateral release and medial capsulorraphy were performed according to protocol [1] and not changed for additional radiographic deformities. Clinical assessment procedures, AOFAS scores, radiological data retrieval, surgical procedures and postoperative treatment are described in detail [1]. The local medical ethics committee approved the study; all patients received oral and written study information and gave their written consent.

### Radiology

Radiological evaluation was performed according to standardized procedures. One examiner measured the HVA and IMA on obtained dorsoplantar x-rays: The HVA was measured as the angle between the line from the center of the metatarsal base to the center of the first metatarsal head and the line connecting the midpoints of the proximal and distal articular surfaces of the proximal phalanx. The IMA was measured as the angle between the line of MT 1 and the line bisecting the diaphyseal portions of metatarsal two [11].

The distal metatarsal articular angle was measured according standard guidelines [12]. Points are placed at the most medial and most lateral extent of the metatarsal articular surface. A line is drawn connecting the two points. Another line is drawn perpendicular on this line. The angle between the perpendicular line and the longitudinal axis of the first metatarsal is the DMAA. The mean of two independent measurements by one reviewer was calculated.

Subluxation of the first MTP joint was classified when the lateral articular border of the proximal phalanx passes the lateral articular border of the first metatarsal.

### Statistics

Data were tested for normality with Levene's test for equality of variances. In case of normal data distribution an independent t-test was used to assess differences between HVA, IMA and DMAA in Chevron versus Scarf osteotomy. Operation results were analyzed by means of logistic regression correcting for age, gender, HVA, IMA and DMAA.

Finally, pre-operative HVA, IMA and DMAA scores were plotted versus postoperative HVA to gain insight into the technical limitations of both osteotomies.

#### Approval study protocol

Medical ethic committee. Maaslandziekenhuis, Sittard: the Netherlands

Protocol was accepted 20 july 1999, code: 99.018

## Results

Clinical improvement according AOFAS score improved from 46 to 87, this was mainly due to reduction of pain, ability to wear shoes and correction. Clinical and radiological pre- and postoperative data were normally distributed between both groups before and after correction of hallux valgus (table 1). Forty cases were included since our findings in a previous randomized controlled trial [1], therefore, current data in table concern an expanded study group with longer follow-up and were not published before. Regression analysis showed a significant influence of preoperative HVA, but no influence from IMA, DMAA, age, osteotomy and gender on hallux valgus angle after surgery.

**Table 1:** Scarf versus Chevron. Normal distribution of data

Variable	Chevron (N = 70)	Scarf (N = 66)	p
Male	9	9	
Female	61	57	
Age	42.0 ± 12.1	45.4 ± 13.1	0.858
Hallux valgus angle			
Preoperative	30.5 ± 6.7	30.0 ± 6.9	0.660
Postoperative	17.2 ± 5.2	19.0 ± 7.7	0.124
Intermetatarsal angle			
Preoperative	13.4 ± 2.4	13.1 ± 2.6	0.490
Postoperative	9.5 ± 2.0	9.4 ± 2.2	0.648
Distal metatarsal articular angle Preoperative	13.0 ± 6.9	12.1 ± 6.8	0.469
Postoperative	12.4 ± 6.3	12.1 ± 6.8	0.803
AOFAS			
Preoperative	46 ± 13.6	47 ± 13.4	0.61
Postoperative	86 ± 20.4	88 ± 14.6	0.38

**Figure 1:**
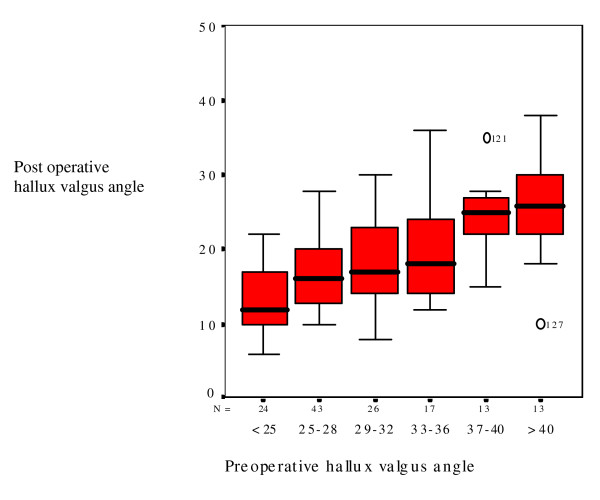
**boxplot: **Preoperative hallux valgus angle versus postoperative hallux valgus angle.

Figure 1 shows the relation between pre- and postoperative HVA. Correction decreased with an HVA of 37 degrees or more. Consequently, HVA of 37 degrees was used as the cut-off point, classifying these cases as severe hallux valgus. Patients (19%) with a preoperative HVA of 37 degrees or more have a worse postoperative HVA than patients (81%) with an HVA of 36 degrees or less (table 2).

**Table 2:** Overall group: HVA <= 36 versus HVA > 36

Variable	HVA < 37 (n = 110)	HVA => 37 (n = 26)
Hallux valgus angle		
Preoperative	27.8 ± 4.6	40.9 ± 3.3
Postoperative	16.5 ± 5.5	25.0 ± 6.8
Change	11.3 ± 5.7	16.0 ± 6.8
Intermetatarsal angle Preoperative	12.8 ± 2.2	14.8 ± 3.0
Distal metatarsal articular angle Preoperative	11.2 ± 6.0	18.3 ± 7.3

**Figure 2:**
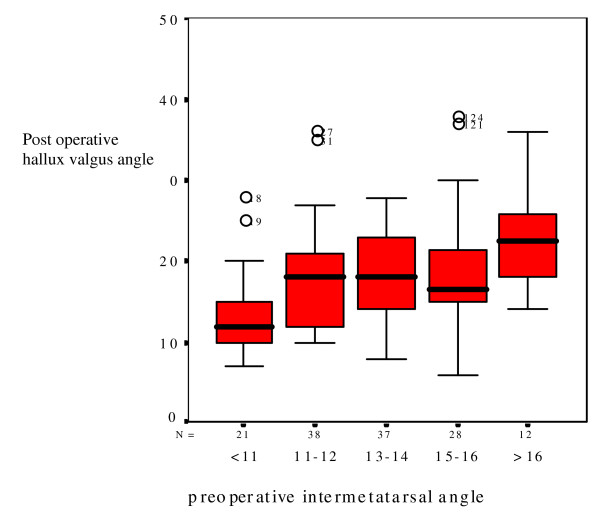
**boxplot:** Preoperative intermetatarsal angle versus postoperative hallux valgus angle.

**Figure 3:**
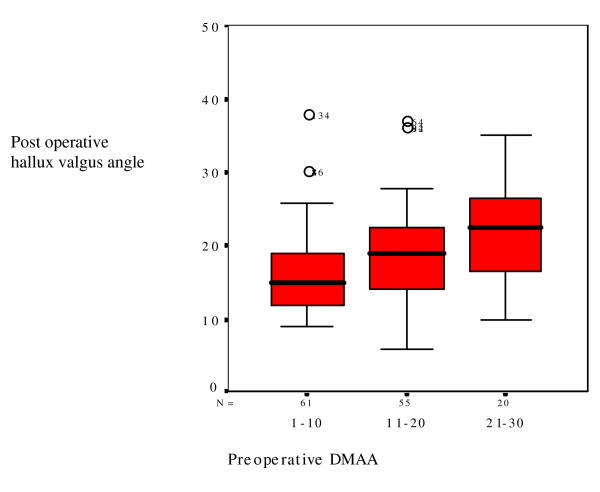
**boxplot**: Preoperative distal metatarsal articular angle versus postoperative hallux valgus angle.

Although IMA and DMAA are not significant in regression analysis, these parameters are increased in patients with HVA of 37 degrees or more (table 2). Therefore IMA is plotted versus postoperative HVA in figure 2, and DMAA is plotted versus postoperative HVA in figure 3. Preoperative IMA suggest a cut-off point of 17 degrees, although there are positive and negative outliers in all groups.

In the severe hallux valgus group 65% had a congruent joint with a mean HVA of 20 degrees that increased 1 degree from 3 months postoperative till last follow-up. The remaining 35% of patients had developed subluxation of the MTPJ with a mean HVA of 32 degrees. Mild and moderate cases tend to keep a constant HVA from 3 months till last follow-up. Severe cases tend to progress after 3 months.

All severe recurrences occurred in cases that developed subluxation of the metatarsophalangeal joint (MTPJ) after initial good correction. These twelve cases were nine percent of the overall group, subdivided by three of 110 cases (3%) with a HVA below 37 degrees, and nine (35%) of 26 cases with a HVA of 37 degrees or more.

## Discussion

We found the preoperative HVA as main predictor for correction of hallux valgus. IMA and DMAA did not predict outcome, although they were increased in severe hallux valgus. In one third of the patients with severe hallux valgus subluxation occured.

Ninety-one percent of the patients were successfully corrected with an osteotomy and transarticular lateral release. The nine percent recurrence of hallux valgus in our study is comparable with reports in the literature [2]. Mann was the first to publish limitations of McBride procedure in cases with high IMA [3], and therefore advised in these cases to combine DSTP with proximal osteotomy. Coughlin combined HVA with IMA in preoperative planning [4]. Cut-off points differ, and the influence of different parameters in surgery is not published. To our knowledge this is the first study that performed regression analysis on different radiographic parameters. Preoperative HVA was found as main predictor for correction of hallux valgus.

We found a cut-off point of preoperative HVA of 37 degrees. IMA and DMAA were mildly increased under 37 degrees, and three of 110 cases developed subluxation of the MTPJ. The indication for used osteotomies and transarticular lateral release can be extended till HVA of 37 degrees. This finding is clinically relevant, because the main parameter for decision-making was tested for these osteotomies. Although distal osteotomy leads to a lower rate of CRPS [1], amount of translation is limited. Proximal osteotomy seem better suited for severe deformities, although possible complications of proximal osteotomy should be thought over [13]. The best treatment for patients with HVA exceeding 37 degrees need to be tested.

In patients with HVA of 37 degrees or more nine of 26 cases developed subluxation of the MTPJ. Likely an open release of the adductor and sesamoid suspensory ligament would have resulted in better correction [14]. True proximal osteotomy or Lapidus procedure can further improve correction. Patients with subluxated MTPJ were offered revision surgery. However, all patients were not motivated for re-operation because they felt pain reduction was adequate and they were able to wear shoes.

The IMA was not a significant predictor for the postoperative hallux valgus angle in logistic regression, but was significantly increased in patients with severe hallux valgus. In figure 2 there seems a cut-off point at IMA of 17 degrees, although outliers are found with lower IMA. Early reports mentioned that higher IMA could be secondary to metatarsal deviation in development of hallux valgus, instead of being a causative factor of hallux valgus [15]. In HVA over 37 degrees, correction of IMA might play a role.

The DMAA was not significant for prediction of the postoperative hallux valgus angle in logistic regression, but was significantly increased in patients with severe hallux valgus. Figure 3 showed that increasing DMAA correlated with increasing HVA, although outliers were found with lower DMAA. Congruency was used to describe subluxated joints after surgery, but because there is no scale for congruency it was not used in logistic regression. We found DMAA could be measured reliably when the metatarsal head has a flat shape. However, in a round metatarsal head it is difficult to assess the medial point of the articular surface. Another factor which influences the medial point of the articular surface is cartilage and joint degeneration after subluxation in the MTP joint [16]. The postoperative DMAA could be confounded in cases with aggressive bunionectomy, which made determination of the medial articular surface more difficult.

Preoperative HVA is the main radiological predictor in correction of hallux valgus. More than 80 percent of the patients can be corrected with osteotomy and capsulotomy. In patients with an HVA less than 37 degrees IMA and DMAA were mildly increased. Therefore, indication for distal osteotomy can be extended and algorithms possibly could be simplified without sacrificing correction. Patients with an HVA of 37 degrees or more had a good correction in 65 percent, the remaining 35 percent developed recurrence of hallux valgus (7 percent of the overall group). Because inter- and intra-observer difference in measuring angles [17], it might be better to use a cut-off range instead of cut-off point. Decision-making for extensive surgery can be preserved for young, high (cosmetic) demand patients. Future research needs to concentrate on patients with an HVA of 37 degrees and more, to clarify the optimal strategy to use open DSTP, correction of DMAA, Akin osteotomy, proximal osteotomy or a combination of these procedures.

## Conclusion

The preoperative HVA is the main radiological predictor for correction of hallux valgus. Correction rate declined in patients with HVA exceeding 37 degrees, caused by subluxation of the MTPJ. IMA and DMAA did not significantly predict possible correction rate, however, these parameters likely do contribute in preoperative HVA of 37 degrees or more.

## Competing interests

The authors declare that they have no competing interests.

## Authors' contributions

ARD: Writing protocol, retrieving and measuring data. Statistical calculation. Writing article. EdV: Review article. J–WKL: Review article, expert opinion. MdWM: Review article. FFD: Writing protocol, surgical procedures + translation of surgical procedures to article. RADB: Statistical calculation and review article. All authors read and approved the final version of the manuscript.

## Pre-publication history

The pre-publication history for this paper can be accessed here:


